# Five Cases of Biliary Peritonitis Due to Bile Leakage From the Gallbladder During Postoperative Hospitalization After Cardiovascular Surgery

**DOI:** 10.7759/cureus.85848

**Published:** 2025-06-12

**Authors:** Akihiro Fujita, Yoshihiro Takemoto, Yuya Tanaka, Takayuki Kawachi, Kimikazu Hamano

**Affiliations:** 1 Department of Surgery and Clinical Science, Yamaguchi University Graduate School of Medicine, Ube, JPN

**Keywords:** acalculous cholecystitis, acute cholecystitis, biliary peritonitis, cardiovascular surgery, gallbladder ischemia

## Abstract

Biliary peritonitis due to bile leakage from the gallbladder is considered a rare clinical entity. However, at our institution, we encountered five such cases between January 2018 and December 2024, all occurring during the postoperative course following cardiovascular surgery. This report presents clinical backgrounds, preoperative and intraoperative findings, and histopathological characteristics of these cases. We present five cases of biliary peritonitis due to bile leakage from the gallbladder. Four cases were acalculous cholecystitis, and three patients lacked abdominal symptoms. All cases developed during hospitalization after cardiovascular surgeries, including total arch replacement (two cases), ventricular septal rupture repair (one case), femoral-popliteal artery bypass (one case), and endovascular aortic repair (EVAR) (one case). Preoperative imaging showed gallbladder wall thinning and reduced contrast enhancement, while intraoperative findings revealed ischemic changes on the gallbladder serosa with bile leakage. Four patients underwent laparoscopic cholecystectomy, and one underwent open surgery. Histopathological examination revealed necrotic changes extending to the subserosal layer or involving the full thickness of the gallbladder wall at the site of bile leakage. This condition is reported to be difficult to diagnose due to minimal abdominal symptoms. In our series, all five cases developed during the postoperative course of cardiovascular surgery but were successfully diagnosed and treated early, resulting in the survival of all patients. Preoperative CT showed advanced atherosclerotic changes in the aorta, with stenosis and calcifications, particularly in the proximal cystic artery. The combination of cardiovascular risk and systemic stress after major surgery may have contributed to the onset of this condition. Recognizing this condition may lead to early diagnosis and appropriate treatment interventions. Although rare, biliary peritonitis due to bile leakage from the gallbladder can occur with notable frequency during the postoperative course of cardiovascular surgery. Understanding its characteristic imaging findings and clinical context is important for early diagnosis and appropriate management.

## Introduction

Biliary peritonitis due to bile leakage from the gallbladder is considered a rare clinical entity [1-3]. Although this condition has been reported as rare, our department experienced five cases of acute cholecystitis presenting with biliary peritonitis due to transudation of bile from the gallbladder between January 2018 and December 2024, all of which occurred during the postoperative hospitalization period following cardiovascular surgery. These cases suggest that this condition may not be as rare as previously thought and may occur with a certain frequency. All five cases developed in the relatively early postoperative period, within 10 days following cardiovascular surgery (3 to 10 days after the primary procedure), and perioperative hypoperfusion and redistribution of splanchnic blood flow were considered potential contributing factors to gallbladder ischemia. In this report, we describe the diagnostic imaging findings and treatment strategies for biliary peritonitis due to bile leakage from the gallbladder, based on our institutional experience with these five cases.

## Case presentation

We present five cases of biliary peritonitis due to bile leakage from the gallbladder. Patient backgrounds are summarized in Table 1, and intraoperative findings are shown in Table 2.

**Table 1:** Characteristics of the patient Four cases were acalculous cholecystitis. Two cases presented with abdominal pain as the chief complaint, while the remaining three underwent imaging studies triggered by recurrent fever or signs of inflammation. Primary disease at admission: The primary diseases requiring hospitalization were Stanford type A acute aortic dissection (two cases), postmyocardial infarction ventricular septal perforation (one case), peripheral arterial occlusive disease (one case), and ruptured abdominal aortic aneurysm (one case). The respective surgeries performed were total arch replacement (two cases), infarct exclusion procedure for ventricular septal perforation (one case), femoral-popliteal artery bypass (one case), and endovascular aortic repair (EVAR) (one case)

Case	Age sex	Primary disease at admission	Surgery for primary disease	Diagnostic trigger	Gallstones	Abdominal pain	Preoperative WBC (×10³/μL)	Preoperative CRP (mg/dL)	Interval from primary surgery to cholecystectomy	Estimated interval from onset to cholecystectomy
1	80 female	Stanford type A acute aortic dissection	Total arch replacement	Evaluation of inflammatory markers and abdominal pain	No	Yes	11.9	18.6	9 days	Same day
2	75 female	Stanford type A acute aortic dissection, cerebral infarction	Total arch replacement	Evaluation of inflammatory markers	No	No	17.1	12.9	10 days	5 days later
3	76 male	Peripheral arterial disease	Femoral-popliteal artery bypass	Evaluation of inflammatory markers and abdominal pain	No	Yes	12.4	29.9	3 days	2 days later
4	79 male	Ruptured abdominal aortic aneurysm	Endovascular aortic repair (EVAR)	Evaluation of inflammatory markers	No	No	21.1	32.5	5 days	Same day
5	80 male	Postmyocardial infarction ventricular septal rupture	Ventricular septal defect closure (infarct exclusion method)	Evaluation of inflammatory markers	Yes	No	10.5	17.7	8 days	Same day

**Table 2:** Operative findings MI: myocardial infarction Preoperative imaging showed decreased enhancement and thinning of part or all of the gallbladder wall. In some cases, the boundary between the gallbladder and pericholecystic fluid was indistinct. These findings correlated with intraoperative ischemic changes on the serosal surface, which appeared greenish or brownish, consistent with bile staining, and bile transudation was suspected from those areas. No overt perforations were identified. All cases were diagnosed as acute cholecystitis with biliary peritonitis due to bile transudation. Surgery and postoperative course: Laparoscopic cholecystectomy was performed in four cases, with the exception of case five. In case five, a patient with severe heart failure and massive pleural effusion following infarct exclusion surgery for post-MI ventricular septal rupture, open cholecystectomy was selected due to concern about respiratory deterioration from pneumoperitoneum. This one patient required tracheostomy (Clavien-Dindo classification grade Ⅲb), but there were no postoperative deaths

Case	Operative method of cholecystitis surgery	Operative time (min)	Blood loss (mL)	Site of bile leakage from gallbladder	Postoperative complications (Clavien-Dindo grade ≥ Ⅲ)
1	Laparoscopic cholecystectomy	80	15	Diffuse on the dorsal side	None
2	Laparoscopic cholecystectomy	116	12	Neck	None
3	Laparoscopic cholecystectomy	140	4	Fundus	None
4	Laparoscopic cholecystectomy	125	10	Diffuse	None
5	Open cholecystectomy	72	9	Neck to body	Yes (sputum retention → tracheostomy)

Patient background

All patients underwent preoperative imaging with CT (contrast-enhanced CT in four cases; noncontrast CT plus MRI in one case due to dialysis) and were diagnosed with acute cholecystitis. The diagnosis of biliary peritonitis due to transudation of bile from the gallbladder was made based on intraoperative findings. Four cases were acalculous cholecystitis. Two cases presented with abdominal pain as the chief complaint, while the remaining three underwent imaging studies triggered by recurrent fever or signs of inflammation.

Primary Disease at Admission

The primary diseases requiring hospitalization were Stanford type A acute aortic dissection (two cases), postmyocardial infarction (MI) ventricular septal perforation (one case), peripheral arterial occlusive disease (one case), and ruptured abdominal aortic aneurysm (one case). The respective surgeries performed were total arch replacement (two cases), infarct exclusion procedure for ventricular septal perforation (one case), femoral-popliteal artery bypass (one case), and endovascular aortic repair (EVAR) (one case).

Preoperative Imaging, Intraoperative Findings, and Resected Specimens

**Figure 1:**
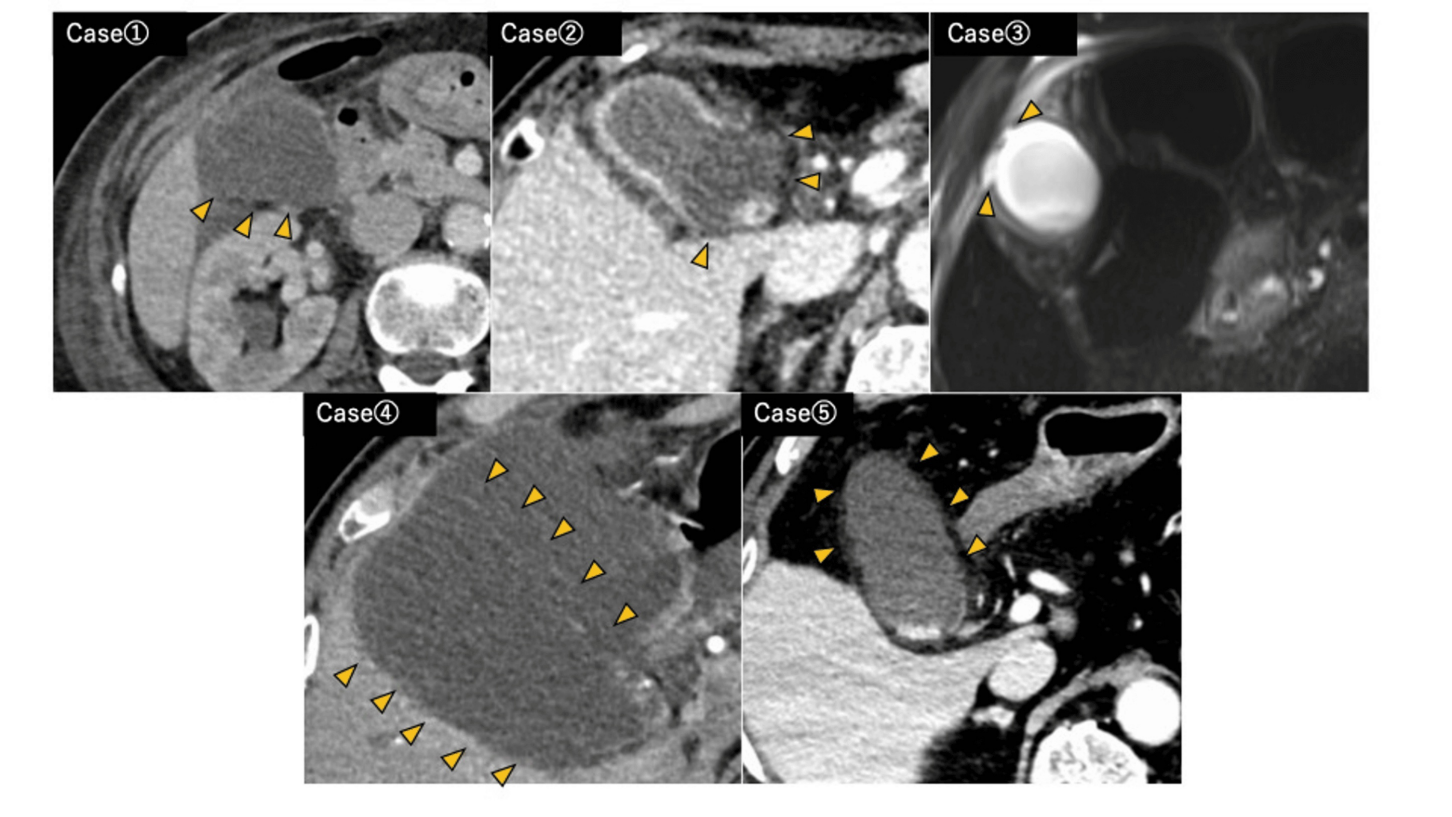
Preoperative CT or MRI findings Cases 1, 2, 4, and 5: Contrast-enhanced CT showing reduced enhancement of the gallbladder wall at the areas indicated by the arrowheads. Case 3: MRI T2-weighted image showing a high-intensity area seen from the gallbladder fundus along the abdominal wall, suggesting bile leakage

**Figure 2:**
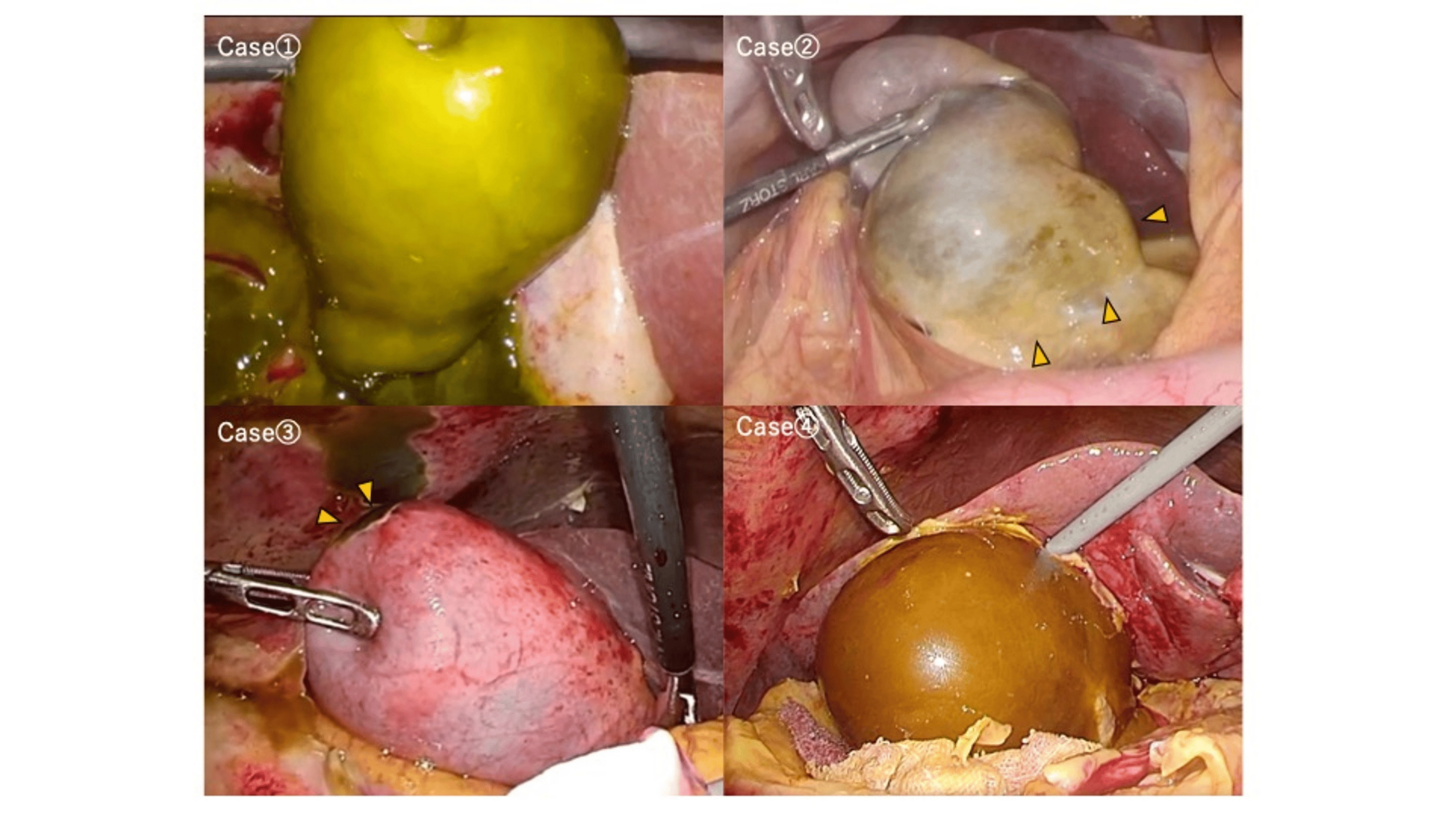
Intraoperative laparoscopic findings in four cases (case numbers correspond to those in Figure 1) Case 1: The gallbladder wall, except for the hepatic bed, appeared greenish in color, and bile accumulation was observed in the surrounding area. Case 2: The gallbladder neck (arrowheads) showed green discoloration, with bile accumulation around it. Case 3: The gallbladder fundus (arrowheads) showed green discoloration, and similar discoloration and adhesion were seen in the adjacent abdominal wall, along with bile accumulation. Case 4: The gallbladder appeared diffusely reddish-brown, with bile accumulation in the surrounding area

**Figure 3:**
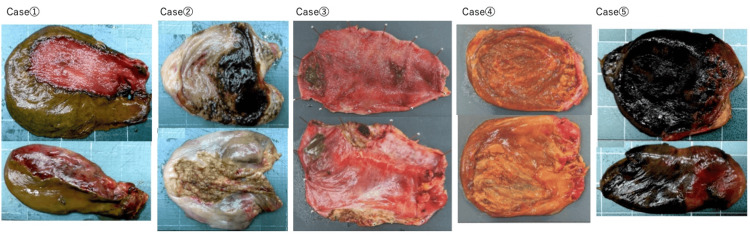
Macroscopic findings of resected gallbladder specimens The upper row shows the mucosal surface, and the lower row shows the serosal surface (case numbers correspond to those in Figure 1). In each case, bile discoloration was observed at the site of contrast effect loss on CT, and bile leakage was confirmed from that area. No obvious macroscopic perforation was identified in any of the five cases

Preoperative CT or MRI findings are presented in Figure 1, intraoperative findings in Figure 2, and resected specimens in Figure 3. Preoperative imaging showed decreased enhancement and thinning of part or all of the gallbladder wall. In some cases, the boundary between the gallbladder and pericholecystic fluid was indistinct. These findings correlated with intraoperative ischemic changes on the serosal surface, which appeared greenish or brownish, consistent with bile staining, and bile transudation was suspected from those areas. No overt perforations were identified. All cases were diagnosed as acute cholecystitis with biliary peritonitis due to bile leakage from the gallbladder.

Surgery and Postoperative Course

Laparoscopic cholecystectomy was performed in four cases, with the exception of case five. In case five, a patient with severe heart failure and massive pleural effusion following infarct exclusion surgery for post-MI ventricular septal rupture, open cholecystectomy was selected due to concern about respiratory deterioration from pneumoperitoneum. This one patient required tracheostomy (Clavien-Dindo classification grade Ⅲb), but there were no postoperative deaths.

Pathological Examination

**Figure 4:**
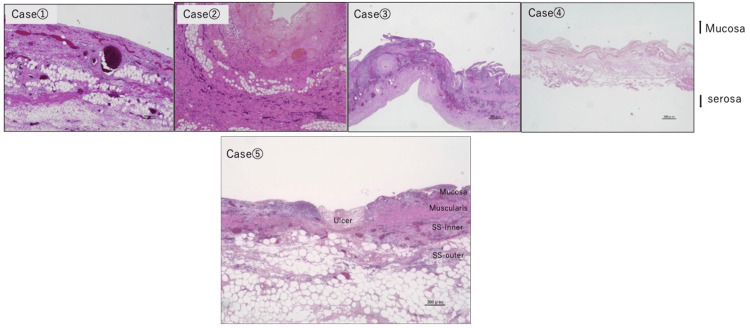
Histopathological findings Case 1-3: Necrosis was observed from the mucosal layer to the SS-outer (fat-rich) layer at the sites of bile leakage. Case 4: The gallbladder wall was markedly thinned and showed full-thickness necrosis, making evaluation of the layered structure difficult. Case 5: Multiple ulcers extending from the mucosal to the subserosal layer were observed

In all cases, the sites of bile leakage exhibited transmural hemorrhage and necrosis, consistent with findings of gangrenous cholecystitis (Figure 4). In one case (case five), multiple mucosal ulcers extending to the subserosal layer were observed. In the remaining four cases, necrosis of the gallbladder wall extending to the subserosal tissue was seen at the thinned areas, or the gallbladder wall structure was indistinguishable due to extensive necrotic tissue. Rokitansky-Aschoff sinuses (RAS) were identified in only one case (case five).

## Discussion

As mentioned in the introduction, biliary peritonitis due to bile leakage from the gallbladder is a rare clinical entity. The earliest report from abroad was by Kent et al. [1]. We conducted searches using PubMed and Elsevier’s Scopus with English terms that have been used in Japanese case reports, such as “biliary peritonitis caused by transudation of bile from the gallbladder,” “biliary peritonitis without perforation,” and “bile leakage from the gallbladder without perforation.” However, we were unable to identify any previously published cases describing this condition. Nevertheless, several reports include instances of biliary peritonitis without evident perforation, suggesting that these cases may have presented with the pathophysiology of biliary peritonitis due to bile leakage from the gallbladder [2-7]. These findings suggest that such cases may occur in routine clinical practice, although the underlying disease concept remains insufficiently recognized.

Gastrointestinal complications following cardiovascular surgery are reported to occur in 0.2-4.6% of cases, with the incidence of cholecystitis ranging from 0.1% to 23% [8-15]. At our institution, among 1,966 cardiovascular surgeries (953 cardiac surgeries and 1,013 vascular surgeries) performed between April 2018 and December 2024, there were 12 cases (0.6%) of acute cholecystitis during postoperative hospitalization, of which five were diagnosed as biliary peritonitis without perforation. Given that our five cases represent a significant portion of the 29 reported in Japan, this condition may occur at a nonnegligible frequency, especially in the postoperative course of cardiovascular surgery.

There are no prior reports discussing systemic vascular conditions as a contributing factor to this disease. However, in all five of our cases, preoperative CT revealed significant aortic calcifications, as well as stenosis and mural thrombi in the celiac or common hepatic arteries. One case (postoperative Stanford A aortic dissection) had an anatomical variant of a replaced right hepatic artery (ReRHA) arising from the superior mesenteric artery (SMA), with SMA origin compressed and narrowed by a false lumen. These findings suggest that stenosis and mural thrombi in the proximal cystic artery may be risk factors.

Regarding the pathogenesis of this condition, Kent et al. proposed that acute cholecystitis develops due to cystic duct obstruction by gallstones and that subsequent overdistension of the gallbladder leads to ischemic changes in the gallbladder wall, resulting in increased permeability to bile [1]. In Japanese reports, additional hypotheses have been proposed, including (i) bile leakage from RAS located in thinned areas of the gallbladder wall; (ii) ischemic changes in the gallbladder wall due to arteriosclerosis or venous thrombosis; and (iii) chemical irritation of the mucosa caused by regurgitation of pancreatic juice into the gallbladder secondary to pancreaticobiliary maljunction [16]. In our case series, acalculous cholecystitis was observed in four of the five patients. Histopathological examination revealed no apparent RAS in these four cases; however, RAS was identified in the remaining case (case five). In this particular case, it is possible that bile leakage occurred from the RAS located in a thinned portion of the gallbladder wall. In all five cases, bile leakage occurred in areas of gallbladder wall necrosis extending to the subserosal layer (in case four, full-thickness necrosis precluded identification of layered structures). Histologically, the gallbladder serosa is composed of a monolayer of mesothelial cells. In the presence of inflammatory mediators such as bradykinin and histamine, the permeability of mesothelial cells increases [17]. Therefore, in cases where necrosis of the gallbladder wall extends from the mucosa to the subserosal tissue, bile may leak into the peritoneal cavity even in the absence of an obvious perforation. Moreover, the ischemic changes in the gallbladder wall varied from localized to diffuse cases, suggesting that the previously mentioned mechanism alone may not sufficiently explain the pathophysiology. Although some necrotic areas showed fibrinoid thickening and relatively fresh thrombi in small vessels, no organized thrombi were noted, making microembolism unlikely. A comparable ischemic condition without major arterial obstruction is nonocclusive mesenteric ischemia (NOMI), which arises from sympathetic vasospasm due to systemic hypoperfusion and blood flow redistribution [18,19]. In our cases, patients had underlying cardiovascular diseases and showed atherosclerotic changes in the peripheral arteries of the gallbladder wall. Since biliary peritonitis occurred early postoperatively (within three to ten days), we speculate that perioperative hypoperfusion and altered distribution of splanchnic blood flow may have contributed to decreased gallbladder blood flow, similar to NOMI.

The surgical treatment for acute cholecystitis complicated by biliary peritonitis was successfully performed in four of the five cases in our series, as laparoscopic cholecystectomy had been planned and completed. This was made possible by considering the possibility of this condition preoperatively, which allowed for early diagnosis and timely intervention. These four cases underwent early surgery, with a mean operative time of 106 ± 29 minutes, and no perioperative complications related to the cholecystectomy were observed.

At our institution, we proactively perform laparoscopic cholecystectomy for acute cholecystitis even in early postoperative cardiovascular cases if the patient’s condition permits. This is because prosthetic graft or valve infections carry high mortality (15-38.5%) [20,21], and mediastinitis caused by postoperative cholecystitis has also been reported [22]. Early cholecystectomy is thus considered favorable in such patients.

A notable feature of this disease is the paucity of abdominal symptoms, which may delay diagnosis. In many of our cases, the gallbladder and leaked bile were covered by the omentum or transverse mesocolon, localizing the inflammation and preventing spread to the anterior abdominal wall. Moreover, analgesics and antipyretics commonly used postoperatively may have masked symptoms. Recognizing these characteristics is critical for early imaging and diagnosis.

A limitation of this report is that our department is a cross-disciplinary surgical unit, which may have led to a selection bias toward cases occurring after cardiovascular surgery. While our case series is limited in number, all five patients developed biliary peritonitis due to bile transudation in a similar postoperative context, with consistent clinical and pathological features. These findings may have important clinical implications and support the need for further case accumulation to clarify their clinical significance and underlying pathophysiology.

## Conclusions

Biliary peritonitis caused by transudation of bile from the gallbladder is a rare condition, but it may occur in patients following cardiovascular surgery. In our case series, early diagnosis and timely intervention enabled favorable outcomes with minimally invasive surgical approaches. Recognizing this condition and its characteristic imaging findings is essential for prompt diagnosis and management.
